# Deep two-photon voltage imaging with adaptive excitation

**DOI:** 10.21203/rs.3.rs-5434919/v1

**Published:** 2024-12-13

**Authors:** Shitong Zhao, Eric Hebert, Anna Gruzdeva, Deepthi Mahishi, Hazuki Takahashi, Sungmoo Lee, Yukun A. Hao, Michael Z. Lin, Nilay Yapici, Chris Xu

**Affiliations:** 1 School of Applied and Engineering Physics, Cornell University, Ithaca, NY, USA; 2 Department of Neurobiology and Behavior, Cornell University, Ithaca, NY, USA; 3 Department of Neurobiology, Stanford University, Stanford, CA, USA; 4 Department of Bioengineering, Stanford University, Stanford, CA, USA

## Abstract

Optical imaging of neuronal voltage dynamics is invaluable to studying brain functions. However, high-speed imaging at significant depth is challenging due to the limitations of the short pixel dwell time and the maximum permissible excitation power in tissues. We report high-speed, deep voltage imaging by applying adaptive excitation, which illuminates the regions of interest only. We imaged neuronal voltage activities across two planes down to 500–630 μm depth in the awake mouse brain. Our techniques can be straightforwardly applied to typical two-photon microscopes.

Optical monitoring of electrical activity in individual neurons is critical for understanding the neural dynamics within the brain^1–11^. As neuronal voltage dynamics occur at a time scale of milliseconds, near-kilohertz imaging speed with subcellular resolution is required and has been demonstrated with widefield microscopy^8,9^, confocal microscopy^2,7^ and two-photon microscopy (2PM)^1,3,4,6,10,12^. However, the thermal damage induced by high laser power^13^ limits the photon flux necessary for imaging at high frame rates deep in tissues^14^. While 2PM can image down to 600–700 μm depth in intact mouse brains^15,16^, the best performance of voltage imaging reported so far is limited to monitoring either layer 2/3 neurons^4,12^, or just a single neuron in layer 5 (at ~ 400 μm depth)^3,6^. Furthermore, some of these existing methods rely on innovative yet complicated beam multiplexing schemes^4,12^, risking crosstalk in the signal collection and limiting the voltage recording quality. Random-access multiphoton (RAMP) microscopy using acousto-optic deflectors (AOD) has demonstrated its capability in voltage imaging^3,6^, but only in very few neurons simultaneously due to the limited modulation bandwidth of the AOD. Here, we present a straightforward 2PM method that enables noninvasive voltage imaging of multiple neurons simultaneously across two independent imaging planes deep in the mouse brain (500–630 μm) while still operating below the tissue heating damage threshold. Our method integrates an improved adaptive excitation source (AES)^17^ into a polygon-galvanometer scanning two-photon (2P) microscope and can be readily adopted by biological labs with 2P microscopes. We also show that our technique approaches the performance limit of 2P voltage imaging for the genetically encoded voltage indicator (GEVI) used.

AES has previously been proposed and demonstrated with 2PM for calcium imaging^17^. By allocating all permissible laser power to regions of interest (ROIs) when scanning, AES takes advantage of the small volume fraction of the ROIs in the mouse brain. The concept of AES is illustrated in Figure 1a–b. Briefly, a high-resolution structural image of the sample is obtained by raster scanning at low power and averaging over many frames. In the structural image, ROIs are drawn to encompass the features for the study. For neuronal activity imaging, the ROIs are selected as ovals slightly larger than the labeled neurons to compensate for moving artifacts (Supplementary Note 1). Based on the ROI information, an arbitrary waveform generator (AWG) drives a Pockels cell, which performs direct intensity modulation of a high-power pulsed laser. The Pockels cell is driven to transmit the laser pulses only inside the ROIs but block the laser elsewhere, achieving illumination of only the ROIs with both high pulse rate and energy. Therefore, the AES achieves high fluorescence signal gains by illuminating the ROIs only without increasing the average laser power on the sample, allowing for deep and fast 2P imaging.

We incorporated AES into a 2P microscope equipped with a 72-facet polygon scanner for the fast axis and a galvanometer scanner for the slow axis (Figure 1c). With an optical scan angle of 10 degrees (peak-to-peak), a maximum line rate of 66 kHz, and a clear aperture of 2.2 mm, this polygon scanner enables imaging of a 365 μm linear field-of-view (FOV) at 416 × 416 pixels per frame at ~150 Hz frame rate with subcellular spatial resolution (Supplementary Figure 1). The frame rate can be increased by scanning a rectangular FOV, e.g., 599 Hz frame rate for 416 × 105 pixels per frame or 365 μm × 92 μm. The capabilities of our fast-scanning microscope, combined with the advantage of the AES, enabled deep and high-speed imaging of voltage activity in the mouse brain.

We performed *in vivo* 2P voltage imaging of neurons expressing the GEVI ASAP5^18^ in the visual cortex of awake mice. With the signal gain provided by the AES and near-kHz frame rate of the microscope, we detected fast fluorescence variations corresponding to both suprathreshold and subthreshold voltage activities in individual layer-5 and layer-6 neurons (Figure 2, Supplementary Figures 2–4). Figure 2a shows the substantial difference between imaging at high speed with and without AES. Although the same average laser power (96 mW) was incident on the brain surface at the same location, the fluorescence traces from non-AES imaging suffer from low numbers of collected fluorescence photons/neuron/frame, and thus a very poor signal-to-noise ratio (SNR). The fluorescence time traces of the same neurons using AES imaging show a 40- to 50-fold increase in photons/neuron/frame, resulting in the high-quality recording of the neural spiking dynamics (SNR > 9). To obtain fluorescence traces of similar SNR with conventional 2PM without AES, the laser power on the sample would need to increase by 6.5 to 7 times (~625 mW), well beyond the tissue heating damage threshold^13^. This comparison demonstrates that AES is necessary and capable for deep, high-speed imaging. Using AES and an average laser power of 153 mW, we successfully recorded the voltage activity in multiple neurons simultaneously in L6, at 628 μm beneath the dura (Figure 2b). Fewer lines scanned allowed us to resolve the voltage activities with higher temporal resolution without compromising the SNR at a similar depth (635 μm, Figure 2c). To enhance the functionality of the microscope, we split the imaging laser into two temporally multiplexed ports and directed them to image two FOVs with independently adjustable locations (Figure 1c). We performed simultaneous dual-plane imaging of multiple neurons in L5 across an axial separation of 80–115 μm, with minimal signal crosstalk between the two planes (Figure 2d, Supplementary Figures 2–3). Similar to the findings in superficial layers^7,8^, we observed temporally coordinated subthreshold voltage oscillations across neurons in deep layers and across a 90-μm axial separation (Supplementary Figure 4).

The dual-port imaging (Figure 2d, Supplementary Figures 2–4) with temporal pulse multiplexing at 183 MHz repetition rate approaches the limit posed by the fluorescence lifetime of GFP-based fluorophore. We measured ~18% signal crosstalk between the two imaging planes in our dual-plane imaging data (Supplementary Figure 2b). Such crosstalk is consistent with a fluorescence lifetime of ~ 2.9 ns for ASAP5 and a system impulse response time of ~ 1.8 ns. For comparison, the fluorescence lifetime of GFP is ~ 3 ns^19^. We note that ROIs in different planes usually do not overlap, i.e., the AES patterns for the ROIs of the two planes usually do not overlap temporally. Therefore, the fluorescence crosstalk between the two planes has a negligible impact on our data.

Compared to the previous AES^17^, this work demonstrates a significant improvement in performance with a substantially simpler scheme. Our implementation achieved ~ 6 times higher the laser burst rate (i.e., from 32 MHz to ~ 183 MHz after multiplexing). Direct modulations of a commercial fiber laser by a Pockels cell enabled straightforward application of AES to a typical 2P microscope. Instead of internal modulations of the laser pulses before amplification, as done in the previous demonstration of AES, direct modulations of the laser allowed us to circumvent the challenging step of gain equalization, and the AES can now accommodate any ROI patterns across two planes with no restriction on the spatial distribution of the ROIs, greatly improving the applicability of AES. Our approach also utilized the full power budget allowed by the biological sample for voltage imaging (Supplementary Table 1), demonstrating the full benefit of the AES. Targeted illumination for 2P imaging can also be implemented using spatial light modulators such as digital micromirror devices (DMDs)^20^; however, the use of DMDs not only requires additional complex optical components within the microscope, but also reduces the power throughput and potentially compromises the spatial profile of the excitation beam^20^. Furthermore, simultaneous multi-plane imaging, as shown in this paper, or fiber delivery (e.g., Mini2P^21^) is incompatible with DMDs.

There are techniques to enlarge the imaging FOV of the polygon scanning microscope^22^; however, deep and fast imaging with a large FOV is fundamentally limited by the required SNR and the maximum permissible power in the tissue^14^. Given the performance of the indicator and the required SNR, the maximum number of neurons imaged decreases exponentially as a function of depth. Since the AES imaged the ROIs only, and the full power budget was utilized to obtain the desired SNR (~ 6 on average), the results presented can be used to estimate the performance limit of 2P voltage imaging in terms of imaging depth and the number of neurons imaged. Supplementary Note 2 shows that the results presented here are within approximately a factor of 2 of the theoretical limit of 2P voltage imaging. Indeed, our results highlighted the greatest depth for non-invasive optical voltage recordings of multiple neurons, as well as the first demonstration of dual-plane voltage imaging in L5.

Imaging deeper than 700 μm may favor three-photon (3P) imaging^16,23^, depending on the labeling density of the sample. A 3P AES at 1300 nm is not yet available (e.g. 90 MHz burst rate with > 100 nJ pulses); however, even with such an AES, theoretically only ~ 3 ASAP5-expressing neurons could be imaged at 800 μm depth before reaching the tissue heating damage threshold, assuming the 2P and 3P action cross section ratio for ASAP5 between 920 nm (2P) and 1300 nm (3P) is similar to the one reported for GFP-based calcium indicators^16^ (Supplementary Note 2).

Improving the excitation source will further improve the imaging results by approximately another factor of 2 and essentially reach the performance limit of 2P voltage imaging. Our imaging data was obtained at 3 laser pulses per voxel, which is slightly less efficient than the optimal one-pulse-per-voxel regime^24^. By increasing the laser pulses at the focus ~ 1.7 times and decreasing the repetition rate ~ 1.7 times (which requires a laser with an average output power of 5.5 W and a repetition rate of 54 MHz at 920 nm), our method could reach the limit of imaging > 15 neurons in L6 (~ 650 μm depth) while achieving the one-pulse-per-voxel regime (Supplementary Note 2). Much higher pulse energies will lead to saturation of the fluorophore^25^. The same improvement in the laser would also facilitate simultaneous three-plane imaging of a total of ~ 40 neurons in L5 (~ 500 μm depth) when the pulses are temporally multiplexed by three to a repetition fate of ~ 162 MHz.

Although this work employed ASAP5 as the indicator to demonstrate deep and high-speed imaging, the concept of AES is applicable to imaging other voltage and calcium indicators as well as studying other biological phenomena, and the benefits brought by AES can represent a combination of reducing laser power in tissues, increasing imaging depth, improving imaging speed, and enlarging the imaging FOV. Additionally, the AES can be practically integrated with all conventional laser scanning microscopes as well as several state-of-the-art 2P/3P microscopes^26,27^, without the need to modify existing hardware and software. Therefore, the AES approach demonstrated here has the potential for straightforward translation to the broad imaging communities, pushing the limits of imaging depth, speed, and FOV.

## METHODS

### Dual-plane AES.

**Figure 1.**
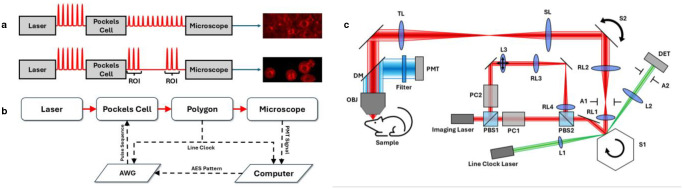
The dual-port AES-polygon microscope. (**a**) Concept of AES. A high-resolution structural image is first obtained using raster scanning at low laser power. The ROIs are defined in the structural image, and the Pockels cell modulates the laser pulses so that only the ROIs are illuminated during scanning. (**b**) Block diagram of the polygon scanning microscope. The line clock from the polygon is shared with the AWG and the computer to ensure synchronization between image acquisition and laser modulation by the AES pattern. AWG, arbitrary waveform generator. (**c**) Schematic diagram of the dual-port AES-polygon microscope. PBS1 splits the beam into two ports, with each port being modulated by a separate Pockels cell to match the illumination patterns for two axially displaced planes. An optical path length difference is also introduced to multiplex the pulse train from 91.4 MHz to 182.8 MHz. The optical pathways of two ports are re-combined by PBS2, and directed to a polygon-galvo scanning microscope. The lens L3 is relayed by RL3 and RL4 to the facet of the polygon S1 and is mounted on a translation stage to allow for fine-tuning of the lateral and axial position of the 2nd-port FOV. A diode laser is used to monitor the line clock of the polygon for synchronization. PBS, polarizing beam splitter. PC, Pockels cell. L, lens. RL, relay lens. A, aperture. S, scanner. DET, detector. SL, scan lens. TL, tube lens. DM, dichroic mirror. OBJ, objective lens. PMT, photo-multiplier tube.

The schematic of the laser scanning microscope is shown in Figure 1c. The 2P excitation laser source is a fiber laser at 920 nm (ALCOR-920–5, Spark Lasers: maximum average power 5.5 W, repetition rate 91.4 MHz, pulse width 110 fs FWHM). A polarizing beam splitter PBS1 (PBS125, Thorlabs) and a half-wave plate HWP (WPH05M-915, Thorlabs) split the beam into two ports that have an optical path difference of 1.64 m before being recombined by another polarizing beam splitter PBS2 (PBS515, Thorlabs), creating a multiplexed laser pulse train at 2 × 91.4 MHz. For the second port, a pair of lenses RL3 and RL4 (both ACT508–500-B, Thorlabs) relay a lens L3 onto the polygon facet. Changing the focal length and the three-dimensional position of L3 then adjusts the imaging plane location of port-2 in all three dimensions relative to that of port-1. To achieve AES, separate Pockels cells (360–80-02, Conoptics) modulate each port of the laser. Based on the user-defined ROI information in the structural image, an AWG (PXI-5422, National Instruments) sends the temporal modulation signal to an amplifier (25D, Conoptics) to drive each Pockels cell to transmit the laser pulses only inside the ROIs (12 to 18 μm larger than the neuron diameter to account for the brain motion) but block the laser elsewhere (see Figure 1a for a single-plane conceptual example). The AWG and the microscope are synchronized with the polygon line trigger (see Microscope setup) to ensure illumination of only the desired ROIs (Figure 1b).

For single-plane imaging, the HWP is rotated to distribute all power to the first port.

### Microscope setup.

For imaging we built a custom microscope (Figure 1c) using a 72-facet air bearing polygon scanner (SA34, Cambridge Technology) for the fast-scanning axis with a maximum line rate of 66 kHz. A pair of achromatic doublets RL1 and RL2 (AC508–100-B-ML and ACT508–200-B-ML, Thorlabs) expand the beam by a factor of 2 and conjugate the polygon scanner S1 to a 5-mm galvanometer scanner S2 (6210H, Cambridge Technology) for the slow-scanning axis. Except for Supplementary Figure 4e, all imaging data was obtained by bidirectionally scanning the galvanometer mirror to reduce the flyback time of the galvanometer mirror. An aperture A1 is placed around the focus of RL1 to prevent transmission of components of the beam reflected off at the joining of two polygon facets. The scan lens SL (AC508–100-C, Thorlabs) and tube lens TL (AC508–150-B-ML, Thorlabs) expand the beam by a factor of 1.5 and conjugate the galvonometer to the back aperture of the water-immersion objective OBJ (XLPLN 25xWMP2, 1.05 NA, Olympus). The 1/e^2^ beam diameter at the back aperture is ~ 6 mm, which equates to an excitation NA of ~ 0.5. The two-photon fluorescence is epi-collected by the objective before being reflected by a dichroic beam splitter (FF705-Di01–25×36, Semrock) through an optical filter (525/25, Semrock) onto the PMT (H7422PA-40, Hamamatsu). The resulting electrical signal goes through a transimpedance amplifier (HCA-400M-5K-C, Femto) held in an electrically shielded box with the PMT before going into the analogue-to-digital converter (VDAQ, Vidrio Technologies). The computer housing the acquisition circuitry uses Scanimage 2023 (Vidrio Technologies) with MATLAB R2023b (Mathworks) software to coordinate operation of the scanners, demultiplexing of the laser pulse train, image formation, and positioning of the mouse with a motorized stage (MP-28, Sutter Instrument). The scanline phase of the polygon scanner is tracked by a secondary monitor laser (BL976-PAG500, Thorlabs) focused (by AC254–100-B-ML, Thorlabs) onto the mirror facet before being recollimated (by LA1608-C, Thorlabs) and scanned across an iris to limit the portion of the facet read onto a silicon detector (PDA100A, Thorlabs). The signal from the detector is used as the polygon scanning line clock, to which the AWG and Scanimage are synchronized.

### Image acquisition parameters.

All acquisition parameters for voltage imaging are summarized in Supplementary Table 1.

### Image processing for structural images.

Structural images were normalized with a linear transform of the pixel values to saturate the greatest 0.2–1% pixels in each image. The ‘Green’ lookup table in imageJ was used to display the data.

### Data analysis for activity recording.

The recorded video with AES was first rigid-motion-corrected by a custom MATLAB program based on cross-correlation between each frame of the video and an average of initial frames. The motion correction program also determines and discards the data when, on rare occasions, the structure of interest (neuron) temporarily moves out of the fixed AES illumination pattern (Supplementary Note 1). The fluorescence signal *F* from the membrane of each cell was extracted, and its rolling median over a 0.5-s window was determined as the baseline fluorescence *F*_*0*_ for each neuron. The inverted *ΔF/F*_*0*_ trace was plotted, where *ΔF = F – F*_*0*_. The collected photon count for each neuron was determined from the pixel values at the cell membrane and a calibration factor. This calibration factor was found by measuring the photon counts and the average pixel values when imaging a uniform fluorescein dye pool sample. To avoid photon stacking error during calibration, the excitation laser power was kept low enough to ensure the measured photon count was less than 5% of the laser repetition rate^16^. The signal-to-noise ratio (SNR) of a fluorescence signal trace was calculated as $\Delta F/\sqrt{F_{0}}$.

### Animals.

C57BL/6J mice (Jackson Laboratories, Black 6, stock no. 000664, females or males, 2–6 months old) were housed in groups of 1–5 before and after surgeries on a 12-h reverse light/dark cycle. All experiments conformed to guidelines established by the National Institutes of Health and have been approved by the Cornell University Institutional Animal Care and Use Committee.

### Animal surgery and *in vivo* voltage imaging of awake mice.

Mice were anesthetized with isoflurane and given the analgesic buprenorphine (intraperitoneally, 0.3 mg per kg of body weight) and Dexamethasone (subcutaneously, 5 mg per kg of body weight). Animals were head fixed onto a stereotaxic apparatus (David Kopf Instruments). A 1-mm-diameter craniotomy was made over the right V1 (AP- 2.7, ML- 2.4 from Bregma). A glass pipette (pulled from 3.5” glass capillaries, Drummond Scientific Company) was backfilled with mineral oil. The glass pipette was mounted into a Nanoject III (Drummond Scientific Company) and used for intracerebral viral injections at various cortical depths (DV −0.75: 200 nL, DV −0.95: 100 nL, DV −1.25: 200 nL). A 1:10 mixture of the following viruses (total injection volume = 500 nL) was used for labeling neurons with the voltage sensor: AAV9-CaMK2a-Cre (volume = 50 nL, stock titer = 2.3 × 10^9^ vg/μL) with AAV9-EF1a-DIO-ASAP5f-Kv (volume = 450 nL, stock titer = 6.6 × 10^12^ vg/μL). After the viral injections, a 4-mm-diameter cranial window was made around the injection site according to the procedures described in previous work^28^. Right after surgery and on the following two days, mice were injected subcutaneously with ketoprofen at a dose of 0.15 mg per kg of body weight. *In vivo* imaging was performed at least three weeks after surgery when mice had recovered and habituated to head fixation. All imaging experiments were conducted on awake, head-fixed mice.

## Supplementary Material

1

## Figures and Tables

**Figure 2.**
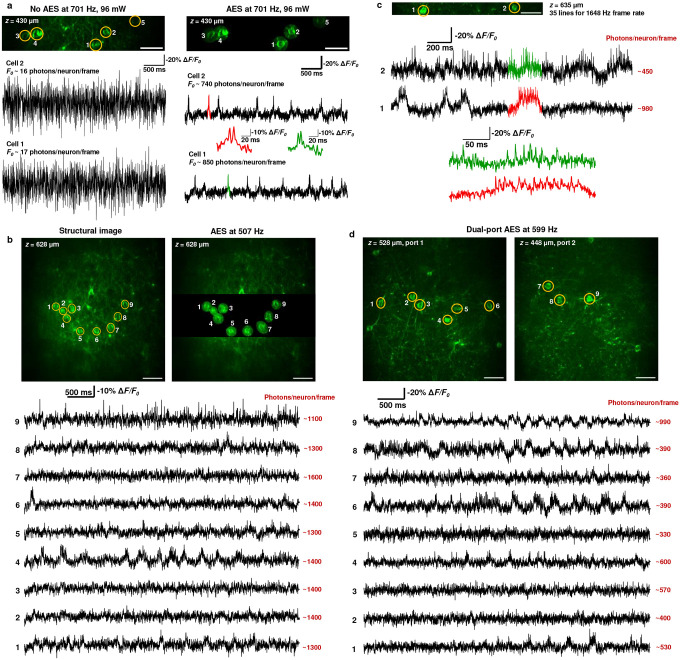
AES enables *in vivo* deep 2PM voltage imaging of multiple ASAP5Kv-expressing neurons in the mouse brain with low excitation power. (**a**) Comparison of voltage recording without (left column) and with AES (right column) at the same location. Both recordings have the same 78 × 365 μm^2^ FOV, 96 mW average laser power, 701 Hz frame rate, and 430 μm imaging depth. Selected portions of traces recorded with AES, colored in red and green, are zoomed in and shown as insets. (**b**) Single-plane optical monitoring of voltage activities in 9 neurons at 628 μm beneath the dura (L6). (**c**) Voltage recording of 2 neurons at 635 μm beneath the dura (L6) at 1648 Hz frame rate. Selected portions of traces in red and green are zoomed in and shown. (**d**) Simultaneous deep (L5), dual-plane voltage imaging by two multiplexed laser ports. Shown in **c** and **d** are structural images without AES. The neuronal activity traces were obtained from AES imaging. The detailed imaging parameters are summarized in Supplementary Table 1. Scale bars, 50 μm.

## Data Availability

The raw data presented in this work is available upon reasonable request to the corresponding authors.
