# Supplementary material for: Deep two-photon voltage imaging with adaptive excitation
Source: Res Sq. 2024 Dec 13:rs.3.rs-5434919. Preprint. [Version 1] doi: 10.21203/rs.3.rs-5434919/v1 (PMC11661313; doi:10.21203/rs.3.rs-5434919/v1)
Supplement: 1 [file NIHPPRS5434919V1-supplement-1.pdf]

Supplementary Figure 1. Microscope characterizations. (**a-b**) Measured 2P response of the microscope for port-1 using a fluorescein dye pool sample. (**c-d**) Measured axial resolution for port-1 by volumetric scanning of a 500-nm thin fluorescent film of Rhodamine B dye. The FWHM of the axial intensity profiles is plotted along the (**c**) slow axis and the (**d**) fast axis. (**e-h**) Measurements for port-2 at the same axial plane as port-1. (**i-l**) Measurements for port-2 when its imaging plane is axially displaced by 135 μm.

Supplementary Figure 2. One-minute video recording of the same site as in **Figure 2d**. (**a**) Structural images at each plane without AES. The grey boxes indicate the rectangular FOVs for high-speed AES imaging. (**b**) Simultaneous dual-plane voltage imaging with AES. Imaging FOV, 92 x 365 μm^2 each plane; frame rate, 599 Hz; average laser power at brain surface, 88 mW from port 1, and 36 mW from port 2; laser effective repetition rate, 9.1 MHz from port 1, and 6.1 MHz from port 2. (**c**) Spontaneous activity traces of neurons labeled in **a**. The grayed-out area indicates when the neurons temporally moved outside of the AES illumination pattern. Scale bars, 50 µm.

Supplementary Figure 3. Additional voltage imaging with > 100 µm axial separation between two planes simultaneously acquired. Selected portions of activity traces in green and red were zoomed in and shown below. Imaging FOV, 110 x 365 μm^2 each plane; frame rate, 507 Hz; average laser power at brain surface, 58 mW from port 1, and 60 mW from port 2; laser effective repetition rate, 5.6 MHz from port 1, and 10.4 MHz from port 2. Scale bars, 50 µm.

Supplementary Figure 4. Synchronized neuronal subthreshold voltage activities observed in deep layers with AES. (**a**) Simultaneous dual-plane neuronal activity recording of 5 neurons located at 480 µm and 6 neurons located at 390 µm beneath the dura. Scale bars, 50 µm. Shown here are structural images at each plane. (**b**) Spontaneous activity traces of the 11 individual neurons labeled in **a**. Grey: raw activity traces. Orange: moving average of activity traces (over 30 ms) highlighting the subthreshold activities. Synchronization of subthreshold oscillations can be detected across different layers. (**c**) Power spectra for each recorded trace in **b**. (**d-e**) Additional single-plane voltage activity recording examples showing synchronized subthreshold oscillations. The black bars at the bottom of traces indicate when a blue LED is turned on as visual stimuli. Scale bars, 50 µm. The three examples above were obtained from three different mice.

Supplementary Figure 5. Phenomenological photobleaching effect with AES imaging. (**a-c**) Raw photon counts data for neurons #4, #8, and #9 as in **Figure 2d** (also **Supplementary Figure 2**). Panel **a** data was obtained without prior high-pulse-energy excitation by AES. Panel **b** data was obtained after the neurons had been imaged with high-pulse-energy excitation by AES by 5.3 s for 5 bouts, with a 1-min interval break between bouts. Panel **c** data was obtained after exposure by 5.3 s for 5 bouts, and by 20 s for 2 bouts, with a 1-min interval break between bouts. The grayed-out area indicates when the neurons temporally moved outside of the AES illumination pattern. (**d-f**) Normalized fluorescence time trace with continuous AES excitation. Plotted here are averages over (**d**) 49 neurons, (**e**) 30 neurons, and (**f**) 15 neurons of the temporal moving averages over 0.5 s for each neuron.

Supplementary Figure 6. Motion correction with AES. (**a**) Diagram describing pre-correction data processing in one AES illumination region along a single line. After the average value within the AES region is subtracted while the unilluminated pixels are set to zero, the dominant effect of the AES pattern on cross-correlation calculations gets eliminated. Rigid motion correction based on cross-correlation is then done on each local AES region. (**b**) The same process of local mean subtraction applied to a frame in an image (top) to produce the frame of comparison for cross-correlation (bottom) used for rigid motion registration. (**c**) Time-averaged projection of a video with motion artifacts before motion correction (top) and after motion correction (middle) alongside the plot of registered displacement over time (bottom). (**d**) Diagrams describing key advantages of motion correction with AES. (top) The relevant features for segmentation, including the neuron (white ring), illuminated region (blue circle), pixels summed for extracting neuron signal (green circle), and extraneous features (red circle). (middle) motion correction ensures that data is collected from pixels of the neuron while excluding fluorescence from nearby unwanted, extraneous features. (bottom) When the neuron drifts outside of the excitation region, signal is lost, which can be flagged with motion registration to reject bad data.

Supplementary Table 1. Imaging parameters for all voltage imaging data in this work. The effective repetition rate is defined as the average number of pulses, at a burst rate of the repetition rate of our laser (91.4 MHz), transmitted to the sample per second to illuminate the ROIs only.

Figure	Imaging depth	FOV	Frame rate	Number of pixels	Post-objective power	Effective repetition rate
2a no AES	430 µm	78 x 365 µm^2	701 Hz	89 x 416	96 mW	91.4 MHz
2a AES	430 µm	78 x 365 µm^2	701 Hz	89 x 416	96 mW	10.9 MHz
2b	628 µm	110 x 365 µm^2	507 Hz	125 x 416	153 mW	9.1 MHz
2c	635 µm	31 x 365 µm^2	1648 Hz	35 x 416	140 mW	8.0 MHz
2d/Sfig2/ Sfig5 a-c	528 µm<br>448 µm	92 x 365 µm^2<br>92 x 365 µm^2	599 Hz	105 x 416<br>105 x 416	88 mW<br>36 mW	9.1 MHz<br>6.1 MHz
Sfig3	530 µm<br>415 µm	110 x 365 µm^2<br>110 x 365 µm^2	507 Hz	125 x 416<br>125 x 416	58 mW<br>60 mW	5.6 MHz<br>10.4 MHz
Sfig4 a-c	480 µm<br>390 µm	110 x 365 µm^2<br>110 x 365 µm^2	507 Hz	125 x 416<br>125 x 416	83 mW<br>55 mW	8.4 MHz<br>9.5 MHz
Sfig4 d	515 µm	78 x 365 µm^2	701 Hz	89 x 416	138 mW	12.8 MHz
Sfig4 e	448 µm	73 x 365 µm^2	532 Hz	83 x 416	87 mW	9.1 MHz

Supplementary Video 1. A video playing the AES data, with motion correction, in **Figure 2a** (right). For visualization, the video has been low-pass filtered with a Gaussian filter of 220 Hz 3-dB bandwidth. The data was acquired at a frame rate of 701 Hz, while the video playback speed is 100 fps. Note the visible activities (blinking) of neurons #1 and #2. Scale bar, 50 μm.

Supplementary Video 2. A video playing the non-AES data in **Figure 2a** (left). For consistency, the video has been low-pass filtered with a Gaussian filter of 220 Hz 3-dB bandwidth. The data was acquired at a frame rate of 701 Hz, while the video playback speed is 100 fps. Scale bar, 50 μm.

Supplementary Video 3. A video playing the AES data, without motion correction, in **Figure 2a** (right). For visualization, the video has been low-pass filtered with a Gaussian filter of 220 Hz 3-dB bandwidth. The data was acquired at a frame rate of 701 Hz, while the video playback speed is 100 fps. Scale bar, 50 μm.

Supplementary Video 4. A video playing the AES data with large motion before correction. Same location as in **Supplementary Figure 4a** (port-2, 390 μm). The data was acquired at a frame rate of 507 Hz, while the video playback speed is 60 fps.

Supplementary Video 5. A movie playing the AES data with large motion after correction. Same location as in **Supplementary Figure 4a** (port-2, 390 μm). The data was acquired at a frame rate of 507 Hz, while the video playback speed is 60 fps.

Supplementary Note 1. Motion correction with AES.

To account for sample motion artifacts, we draw regions of illumination larger than the structures of interest (neurons) and then correct for rigid lateral motion based on cross-correlation between each frame of the video and an average of the first several frames. The AES illumination pattern normally inhibits the ability to register the displacement of the sample because the AES pattern, which does not move with the sample, dominates cross correlations. Using our pre-existing knowledge of the pattern, however, we can eliminate its contribution to motion registration by subtracting the mean value within each AES region while setting all unilluminated pixels to zero (**Supplementary Figures 6a-b**). The resulting corrected video is more stable (**Supplementary Figure 6c** and **Supplementary Videos 4-5**).

Often, we can correct for motion better for AES data than non-AES data because of the improved signal levels. To further reduce the effect of noise, we apply a rolling Gaussian temporal average across adjacent frames before computing the cross-correlation. If the AES regions contain clear features, the added signal per frame allows for fewer frames in the temporal average, allowing us to correct for motions on the order of 20 Hz (**Supplementary Figure 6c**).

The use of motion correction allows us to segment the cell membranes more tightly and accurately during data analysis, eliminating contributions to the signal from features near the neuron which may move into and out of the illumination region as the sample moves (**Supplementary Figure 6d**). Additionally, we can identify and discard the data taken when, on rare occasions, the neuron temporarily drifts outside of the illumination region (**Supplementary Figure 6d**). With the ROI size used in this work (12 to 18 µm larger than the neuron diameter), 5-15 % of the acquired data from awake mouse imaging had to be discarded. For imaging anesthetized mouse, the ROI size can be smaller without losing track of the illuminated neurons.

All procedures described above were carried out using a custom MATLAB program, available through Zenodo (**doi: 10.5281/zenodo.14075134**).

Supplementary Note 2. The limit on the number of measurable ASAP5-expressing neurons by 2P and 3P voltage imaging.

For high-fidelity voltage activity recording, the neuronal fluorescence time traces in this paper, on average, have SNRs of ~ 6 at a frame rate of 500 Hz (**Supplementary Figure SN1**). For the calculations below, we target an average SNR of 6 at a frame rate of 500 Hz. For 2P voltage imaging of ASAP5-expressing neurons, we take 20 % as a typical value for the spike $-\Delta F/F_0$, as commonly observed in our data as well as in ref[1]. These measures indicate that a baseline fluorescence signal F_0 ~ 900 photons/neuron/frame at a frame rate of 500 Hz is required.

The data in **Figure 2b** can be used for calculating the limit on the number of ASAP5-expressing neurons imaged, as close to the maximum permissible power was used in this case. In this dataset, there are on average N ~ 600 pulses per neuron per frame at a burst rate, f_{burst}, of 91.4 MHz impinged on each cell membrane with ~ 0.3 nJ energy at the focus, ε_{2P}, for generating fluorescence signals of F_0 ~ 1300 photons/neuron/frame at ~ 500 Hz frame rate. Therefore, we estimate that the number of detected signal photons per excitation pulse at the focus is

$$\frac{F_o}{N} = \frac{1300\ \text{photons/neuron/frame}}{600\ \text{pulses/neuron/frame}} = 2.2\ \text{photons/pulse}$$

If not limited by the performance of our current laser, the signal photons can be increased further with higher pulse energies, ε_{2P}, up to ~ 0.5 nJ at the focus, beyond which saturation may take place for NA = 0.5 (used in our microscope)[2]. At ε_{2P} ~ 0.5 nJ at the focus, we estimate F_o/N ~ 6.1 photons/pulse. Assuming an excitation power of P_{2P} = 160 mW on the brain surface, and an effective attenuation length of l_{2P} = 160 µm, the maximum effective repetition rate f_{eff}, at a depth of z = 640 µm, can be calculated as

$$f_{eff} = \frac{P_{2P}}{\varepsilon_{2P}\exp(z/l_{2P})} \sim 5.9\ \text{MHz}$$

This maximum effective repetition rate f_{eff} denotes the maximum number of active AES pulses per second; however, to accommodate sample motion during capture, the AES illumination pattern is typically ~ 5 times as large as the donut-shaped area of the cell membrane, over which the pixels are integrated for extracting neuronal activity signals. If we consider only the excitation pulses on the cell membranes, the useful portion of the f_{eff} scales from 5.9 MHz to 1.18 MHz. Therefore, the number of collected 2P signal photons S_{2P} ~ 1.18 MHz x 6.1 photons/pulse = 7.2M photons/s, which allow for imaging $\frac{7.2\ \text{M photons/s}}{900\ \text{photons/neuron/frame x 500 Hz}} = 16$ neurons. This is approximately the theoretically maximum number of ASAP5-expressing neurons that can be imaged with 2PM at 640 µm depth. This number decreases exponentially with imaging depth, as plotted in **Supplementary Figure SN2.** When compared to similar quantitative characterizations of the number of measurable neurons expressing the GEVI JEDI-2P[3,4], our calculations suggest an increase by an order of magnitude in deep layers, primarily due to the preferable feature of the

AES that the effective laser repetition rate can be freely adjusted with imaging depth to maximize the 2P signal[2,5].

For deeper voltage imaging, we extrapolate our calculations to 3PM. We base our extrapolation on a previous quantitative analysis of 2P and 3P calcium imaging by Wang et al.[5]. Theoretically, assuming a Gaussian beam excitation focus, the numbers of signal photons per excitation pulse at the focus for 2P and 3P, respectively, are given by[6]

$$\frac{S_{2P}}{f} = \frac{1}{2}\frac{g_p^{(2)}}{\tau}\phi C(\eta\sigma_2)n\frac{\pi}{\lambda_{2P}}\varepsilon_{2P}^2$$

$$\frac{S_{3P}}{f} = \frac{1}{3}\frac{g_p^{(3)}}{\tau^2}\phi C(\eta\sigma_3)n\frac{2\pi^2}{3\lambda_{3P}^3}\mathrm{NA}^2\varepsilon_{3P}^3$$

where $g_p^{(n)}$ is the nth-order temporal coherence factor, τ is the laser pulse width, ϕ is the system collection efficiency, C is the concentration of the fluorophore, $\eta\sigma_n$ is the n-photon action cross section, n is the refractive index of the medium, λ is the excitation wavelength in vacuum, NA is the numerical aperture defined by the $1/e^2$ beam diameter at the objective back aperture, ε_{2P} and ε_{3P} are the pulse energy at the focus for 2P and 3P in the unit of photon, and f is the repetition rate.

It is reported that for GCaMP6s, it requires ε_{3P} = 1.86 nJ at λ_{3P} = 1320 nm, τ = 60 fs, and NA = 0.75 to generate the same signal level (0.1 collected photon/pulse) for a 3P process as ε_{2P} = 0.24 nJ at λ_{2P} = 920 nm and τ = 60 fs would do for a 2P process[5]. If we assume that the ratio of the 2P and 3P action cross sections for ASAP5 is similar to that for GCaMP6s, then we could speculate that for 3P voltage imaging of ASAP5-expressing neurons, a pulse energy ε_{3P} = 2.3 nJ at λ_{3P} = 1320 nm, τ = 60 fs, and NA = 0.5 (used in our microscope) would maintain the signal level (i.e. $S_{3P}/f = S_{2P}/f$ = 2.2 photons/pulse) achieved with our 2P experiments at ε_{2P} = 0.3 nJ, λ_{2P} = 920 nm, and τ = 110 fs (note the 2P parameters need to be scaled to ε_{2P} = 0.22 nJ and τ = 60 fs). At a depth z = 800 µm, assuming an excitation power P_{3P} = 100 mW on the brain surface and an effective attenuation length l_{3P} = 300 µm, the maximum effective repetition rate f_{eff} of the 3P laser can be calculated as

$$f_{eff} \sim \frac{P_{3P}}{\varepsilon_{3P}\exp(z/l_{3P})} = \frac{100\ \mathrm{mW}}{2.3\ \mathrm{nJ} \times exp(800\ \mu\mathrm{m}/300\ \mu\mathrm{m})} = 3.0\ \mathrm{MHz}$$

Such number of active AES pulses equates to a total 3P neuronal signal of S_{3P} ~ 3.0 MHz x 20 % (cell membrane) x 2.2 photons/pulse = 1.32 M photons/s, which allow for imaging $\frac{1.32\ \mathrm{M\ photons/s}}{900\ \mathrm{photons/neuron/frame\ x\ 500\ Hz}}$ = 3 neurons. The maximum number of ASAP5-expressing neurons measurable as a function of depth for 3P processes is also plotted in **Supplementary Figure SN2.**

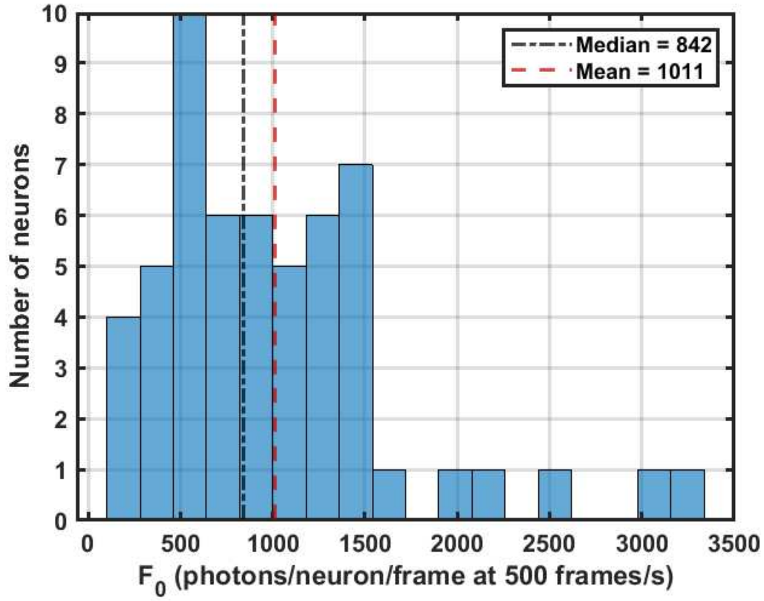

Supplementary Figure SN1. A histogram of baseline fluorescence signals F_0, scaled to a frame rate of 500 Hz, for each neuron reported in this work. The median (black) and the mean (red) of this distribution are marked.

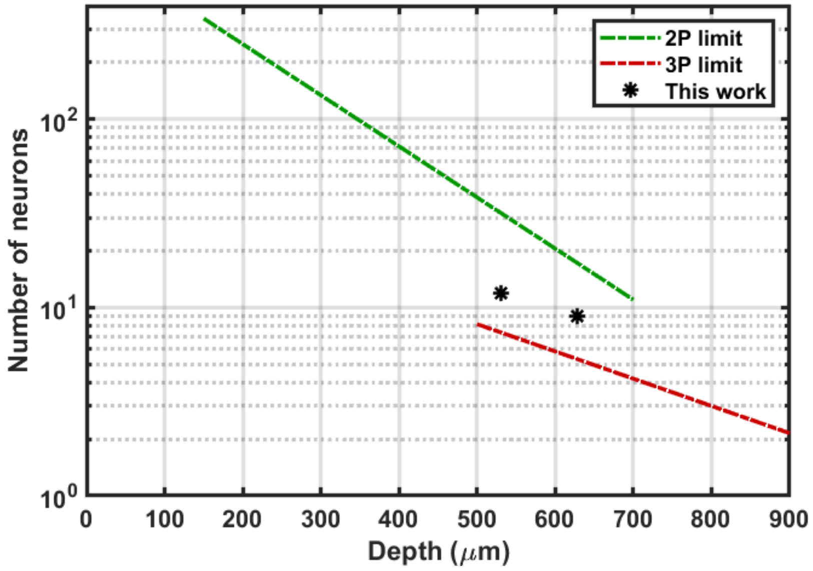

Supplementary Figure SN2. A plot of the theoretically maximum number of ASAP5-expressing neurons measurable as a function of depth for 2PM (green) and 3PM (red) alongside our experimental results (asterisks).

References

1. Hao, Y. A. *et al.* A fast and responsive voltage indicator with enhanced sensitivity for unitary synaptic events. *Neuron* (2024) doi:https://doi.org/10.1016/j.neuron.2024.08.019.
2. Charan, K., Li, B., Wang, M., Lin, C. P. & Xu, C. Fiber-based tunable repetition rate source for deep tissue two-photon fluorescence microscopy. *Biomed Opt Express* **9**, 2304 (2018).
3. Liu, Z. *et al.* Sustained deep-tissue voltage recording using a fast indicator evolved for two-photon microscopy. *Cell* **185**, 3408-3425.e29 (2022).
4. Phil Brooks, F., Davis, H. C., Wong-Campos, J. D. & Cohen, A. E. Optical constraints on two-photon voltage imaging. *Neurophotonics* **11**, (2024).
5. Wang, T. *et al.* Quantitative analysis of 1300-nm three-photon calcium imaging in the mouse brain. *Elife* **9**, (2020).
6. Xu, C. & Webb, W. W. Multiphoton Excitation of Molecular Fluorophores and Nonlinear Laser Microscopy. in *Topics in Fluorescence Spectroscopy* (2002). doi:10.1007/0-306-47070-5_11.

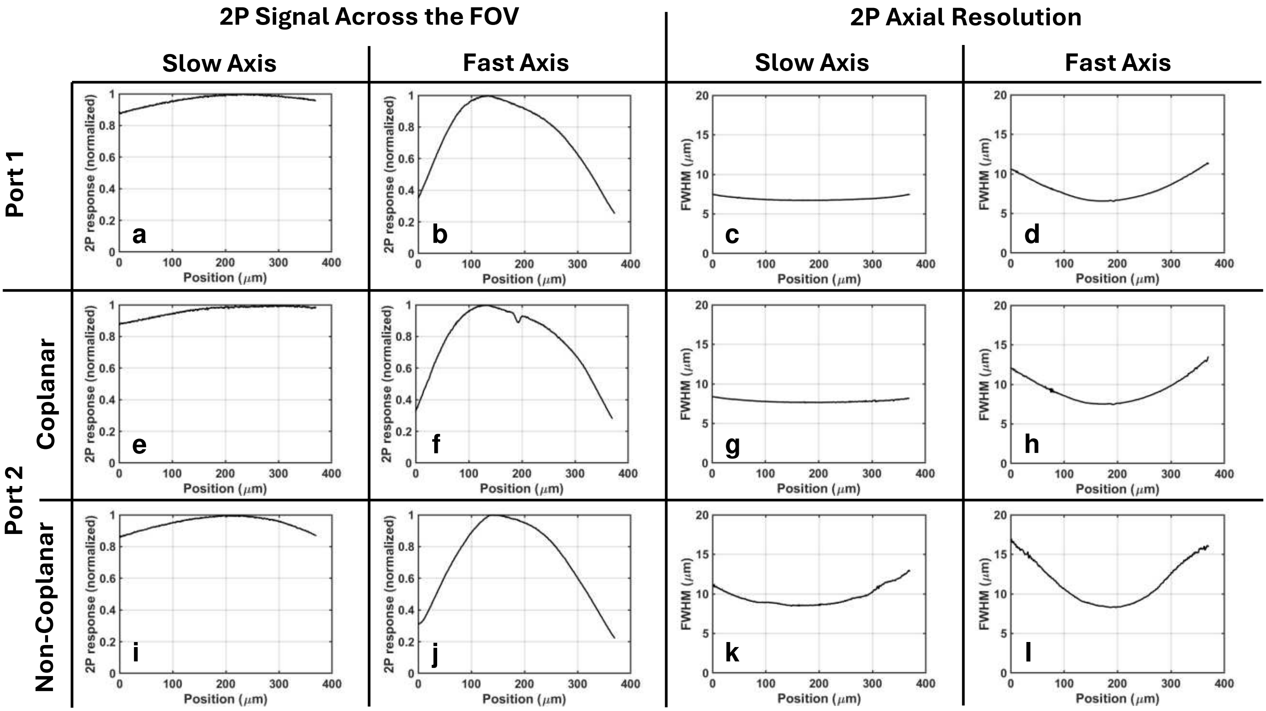

Supplementary Figure 1

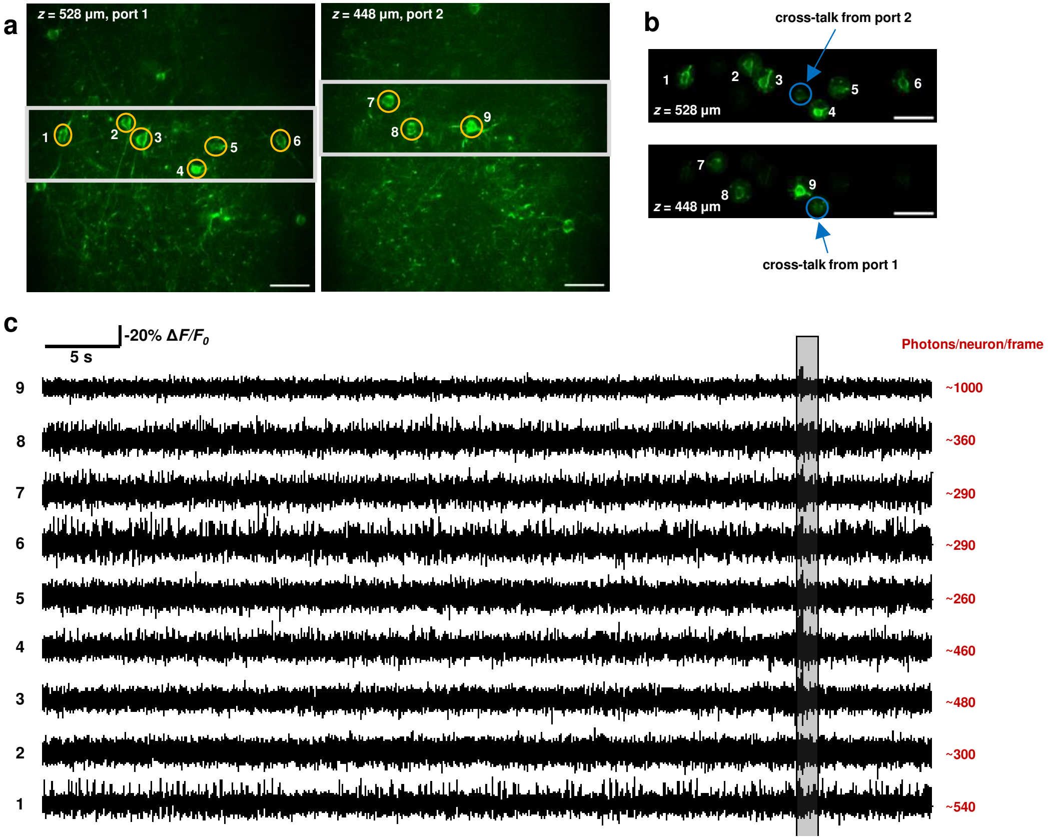

Supplementary Figure 2

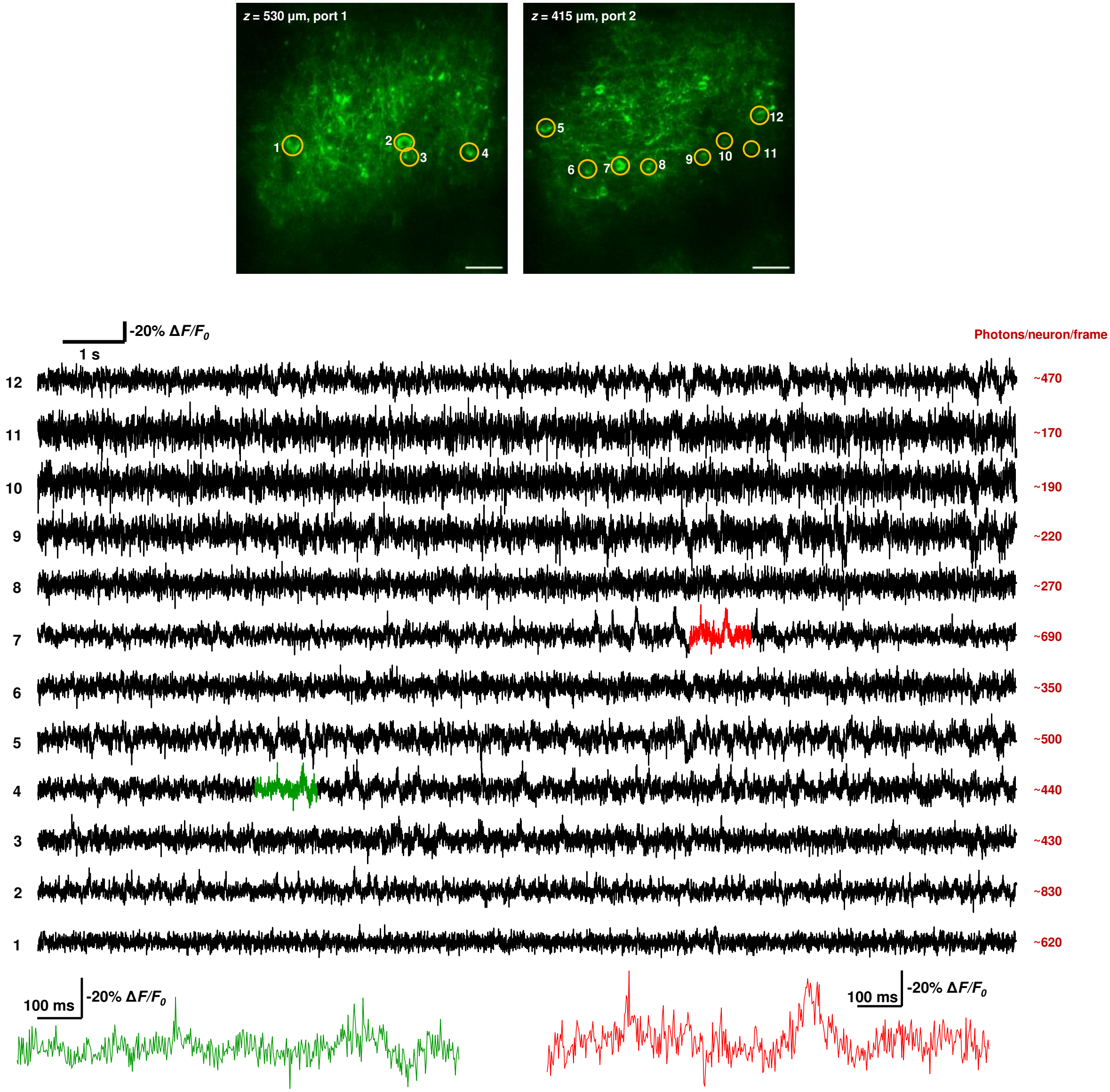

Supplementary Figure 3

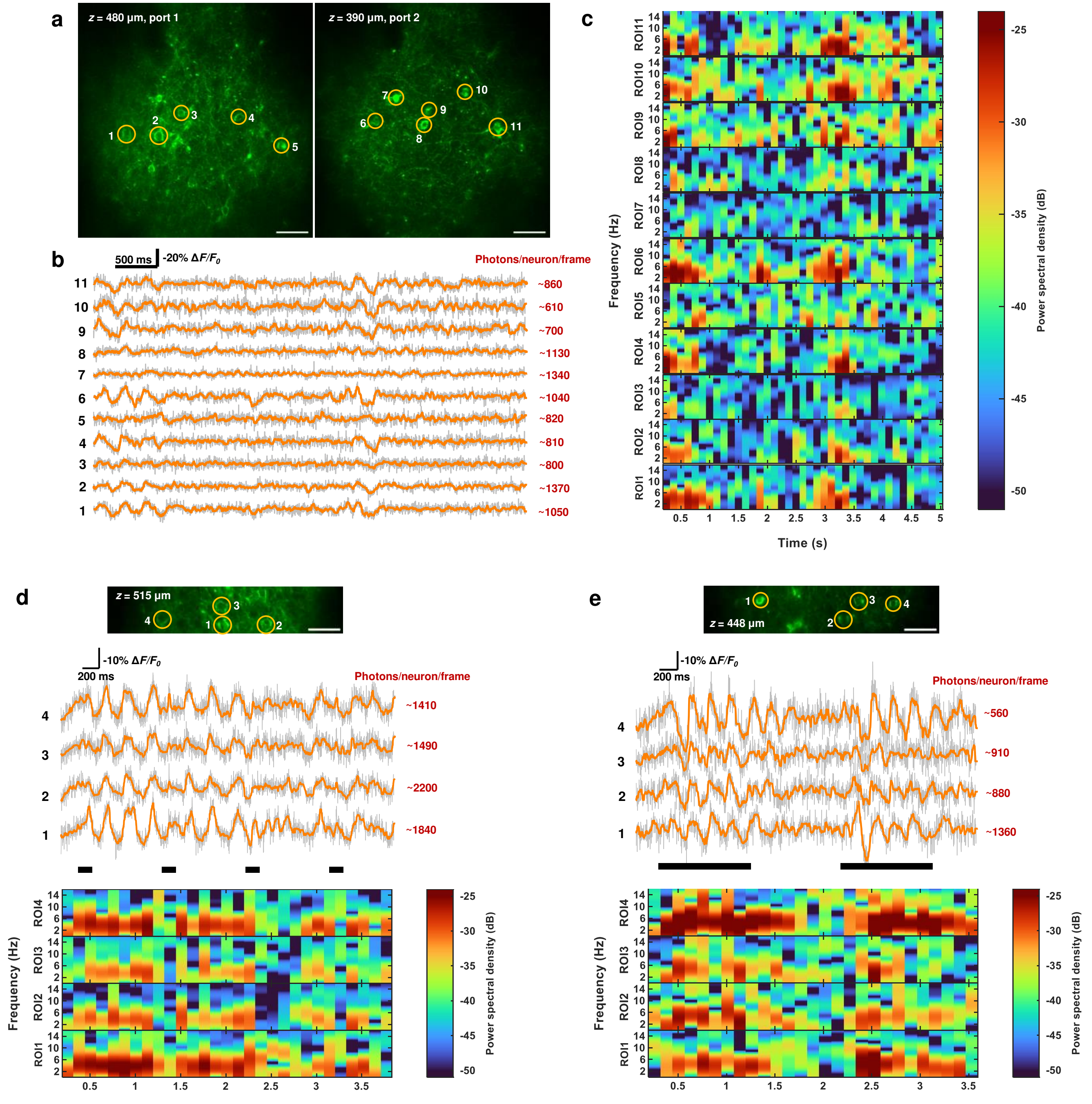

Supplementary Figure 4

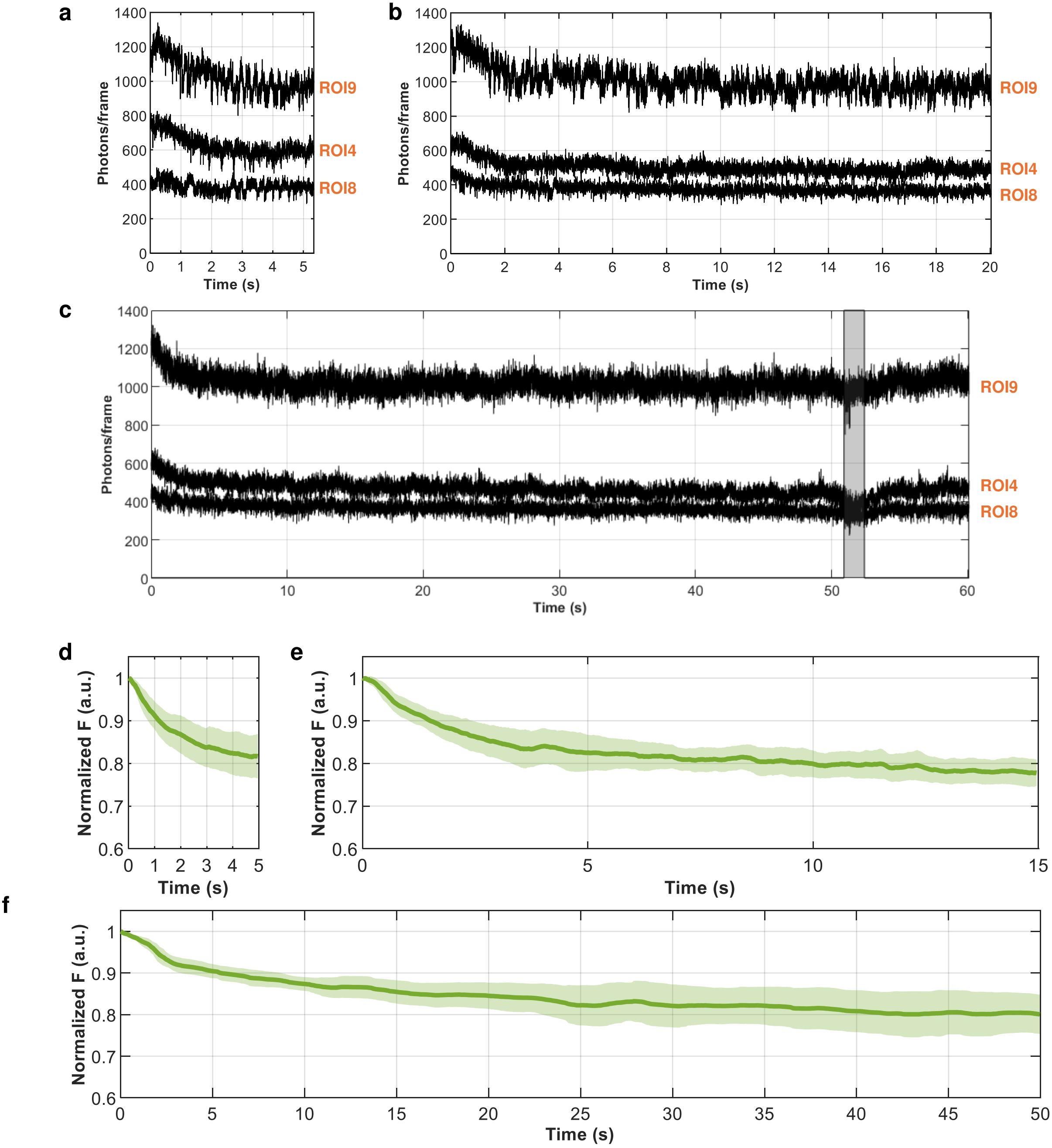

Supplementary Figure 5

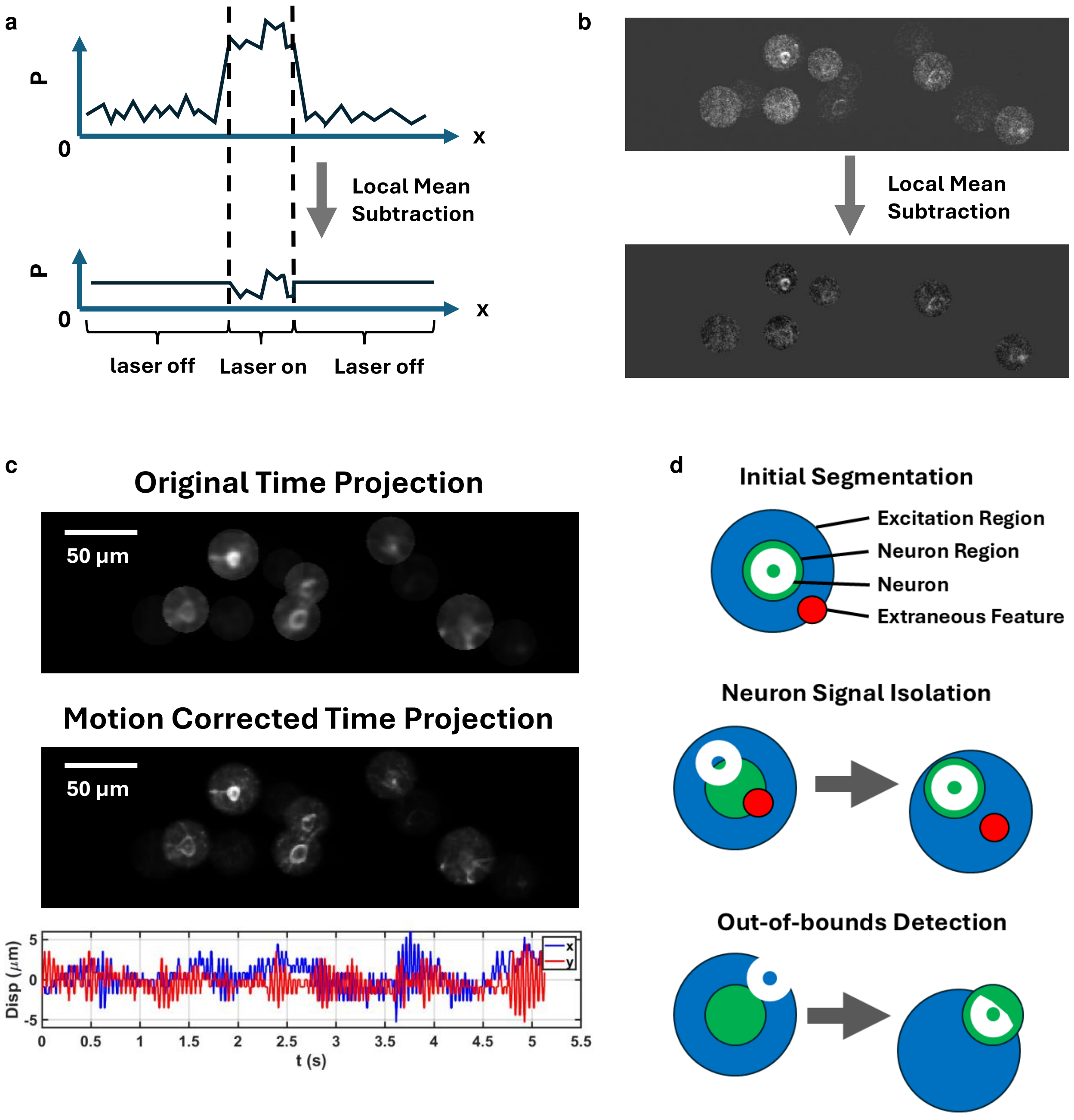

Supplementary Figure 6
